# Hypermethylation of *N*-Acetyltransferase 1 Is a Prognostic Biomarker in Colon Adenocarcinoma

**DOI:** 10.3389/fgene.2019.01097

**Published:** 2019-11-06

**Authors:** Cheng Shi, Li-ye Xie, Yan-ping Tang, Long Long, Ji-lin Li, Bang-li Hu, Ke-zhi Li

**Affiliations:** ^1^Department of Gastroenterology, The People’s Hospital of Liuzhou, Liuzhou, China; ^2^Department of Research, Affiliated Tumor Hospital of Guangxi Medical University, Nanning, China

**Keywords:** methylation, *N*-acetyltransferase 1, colon adenocarcinoma, prognosis, The Cancer Genome Atlas

## Abstract

**Background:** The *N*-acetyltransferase 1 (*NAT1*) gene is downregulated in several cancers and associated with patient survival. In this study, we sought to examine the prognostic value and clinical significance of *NAT1* methylation in colon adenocarcinoma (COAD).

**Methods:** Data relating to *NAT1* mRNA expression and methylation and clinicopathological features of COAD were extracted from the database of The Cancer Genome Atlas. We compared the mRNA expression and methylation of *NAT1* between COAD and normal tissues and performed correlation analysis to assess the association between *NAT1* mRNA expression and methylation. Furthermore, we assessed patient survival based on CpG sites in the promoter region of *NAT1* and analyzed the association between the *NAT1* mRNA expression and CpG site methylation and clinicopathological features. An independent Gene Expression Omnibus (GEO) dataset was used to validate the results.

**Results:** We found that the expression of *NAT1* mRNA was reduced in COAD compared with normal tissues and that mean methylation of the eight CpG sites in the promoter region of *NAT1* was higher in COAD tissues than in normal tissues. Furthermore, five CpG sites were demonstrated to be significantly negatively correlated with *NAT1* mRNA expression in COAD. Survival analysis indicated that *NAT1* mRNA expression and the cg15797286 and cg18509990 sites were associated with the overall survival of COAD patients. Combined survival analysis revealed that combinations of *NAT1* mRNA expression with five CpG sites were significantly associated with the overall survival of COAD patients. Both *NAT1* mRNA and cg15797286 were associated with the T, N, and clinical stages of COAD. The GEO data indicated that cg15797286 was hypermethylated in recurrent colorectal adenomas.

**Conclusions:** Methylation of *NAT1* is associated with the development of COAD, and may serve as prognostic and treatment biomarkers for COAD.

## Introduction

Colorectal cancers (CRCs) are among the most common digestive system malignancies worldwide, of which colon adenocarcinoma (COAD) comprises the majority (Bray et al., 2018). Despite a recent decrease in the incidence of CRC in Western countries, the incidence in those of less than 55 years of age has increased by approximately 2% per year during the past two decades (Siegel et al., 2017). In addition, the burden of CRC in China is considerably higher than that in the USA (Chen et al., 2016). Although numerous studies have examined the mechanisms underlying colorectal carcinogenesis, and despite the fact that certain aspects of the etiology and pathology of CRC are well documented (Baena and Salinas, 2015; Vulcan et al., 2017), many of the associated mechanisms require further elucidation.

DNA methylation is a common epigenetic modification, which may function by silencing gene expression. The aberrant methylation of genes has been demonstrated to play important roles in multiple biological possesses, including DNA repair and apoptosis, and can subsequently lead to carcinogenesis (Saghafinia et al., 2018; Pfeifer, 2018). In addition to environmental factors, the accumulation of multiple genetic and epigenetic alterations or interactions is considered a crucial factor contributing to the pathogenesis of CRC (Jones, 2012; Vogelstein et al., 2013). The aberrant methylation of several genes has been described in previous studies, including that of *HOXA2* (Chen et al., 2019) and *PBX3* (Sun et al., 2019). The association of gene methylation with CRC has also been documented. For example, aberrant methylation of *CDKN2A* has been identified in the breast, prostate, renal, and colon cancers, and has been demonstrated to be associated with transcriptional inhibition (Herman et al., 1995). Furthermore, promoter hypermethylation of *SFRPs* has been shown to inhibit WNT receptor binding, thereby downregulating pathway signaling in CRC (Suzuki et al., 2004), whereas hypermethylation of the *hMLH1* promoter has been found to be related to gene inactivation in sporadic CRC (Cunningham et al., 1998). These findings accordingly indicate that the hypermethylation of certain genes is associated with the pathogenesis of CRC. However, the methylation characteristics of many genes in CRC remain to be elucidated.

*N*-acetyltransferase 1 (*NAT1*) is a phase II xenobiotic metabolizing enzyme that is expressed in almost all human tissues (Hein et al., 2018). In several cancers, the expression of *NAT1* is associated with cell proliferation *in vitro* and with survival *in vivo* (Tiang et al., 2011; Minchin and Butcher, 2018). With regard to CRC, although a previous meta-analysis indicated that the *NAT1* genotype was not significantly associated with an elevated CRC risk (Liu et al., 2012), a further study has demonstrated that *in vivo* knock-down of *NAT1* in the HT-29 COAD cell line promoted an up-regulation of E-cadherin and cell–cell contact growth inhibition, thereby indicating that *NAT1* may be a novel drug target for CRC therapeutics (Tiang et al., 2011). However, little is currently known regarding the methylation of *NAT1* in COAD. Therefore, in this study, we aimed to evaluate the prognostic value and clinical significance of *NAT1* methylation in COAD using RNA-sequence data obtained from The Cancer Genome Atlas (TCGA) database.

## Materials and Methods

### Source of Colon Adenocarcinoma-Related *N*-Acetyltransferase 1 Data

An RNA-seq dataset (Level 3) of COAD was downloaded from the Broad GDAC FireHose website (http://gdac.broadinstitute.org/, accessed June 7, 2019), which includes data for 263 COAD tissues and 30 corresponding adjacent normal colon tissues. We also downloaded DNA methylation profiles for 275 COAD tissues and 39 adjacent normal colon tissues (Illumina Human Methylation 450K) from the Broad GDAC FireHose site. Our use of TCGA data in the present study complied with the TCGA data usage policy.

### Data of *N*-Acetyltransferase 1 mRNA Expression and Methylation of CpG Sites in Cancers

According to data obtained from the MethHC website (http://methhc.mbc.nctu.edu.tw) (Huang et al., 2015), the *NAT1* gene has nine variants, each of which has a unique promoter and certain differences with respect to CpG sites (**Table S1**). However, only the NM_001160179 variant has a significantly higher methylation value (mean beta value > 0.5) in COAD tissues compared with normal tissues (P < 0.001) (**Figure S1**). We thus selected the NM_001160179 variant of *NAT1* for subsequent analysis. We analyzed the expression of *NAT1* mRNA in multiple cancers from the TCGA database using the GEPIA websites (http://gepia.cancer-pku.cn/) (Tang et al., 2017), and extracted and visualized the data relating to CpG sites in the *NAT1* promoter region using the MethHC website.

### Analysis of the Correlation Between *N*-Acetyltransferase 1 mRNA Expression and Methylation Values

We determined correlations between *NAT1* mRNA expression and the mean methylation of *NAT1* promoter CpG sites in COAD using Pearson correlation analysis, and subsequently examined the correlation of *NAT1* mRNA expression with methylation at each of the promoter CpG sites. A P-value of less than 0.05 was considered indicative of statistical significance.

### Analysis of Colon Adenocarcinoma Patient Survival Related to *N*-Acetyltransferase 1 Gene Expression and CpG Site Methylation

Initially, we applied Kaplan–Meier curve analysis and a Log-Rank test to examine the association between *NAT1* mRNA expression and overall survival (OS) in COAD patients, and subsequently performed similar analyses for associations between methylation at each of the *NAT1* promoter CpG sites and OS in COAD patients. Finally, we combined data for *NAT1* mRNA expression and methylation at each of the CpG sites and analyzed the associations between these combinations and patient survival. Cox regression analysis was employed to assess independent indicators associated with the prognosis of COAD patients. Data were analyzed using R language (version 3.5.1) with a P-value of less than 0.05 indicating statistical significance.

### Association Between *N*-Acetyltransferase 1 CpG Site Methylation and the Clinicopathological Features of Colon Adenocarcinoma

The clinicopathological features of COAD patients, including patient age, gender, TNM stage, and clinical stage, were extracted from the clinical data of COAD patients downloaded from the Broad GDAC FireHose website. The *NAT1* mRNA expression and CpG sites that were found to be significantly associated with OS in COAD were used to analyze associations with the clinicopathological features of COAD. Data were analyzed using SPSS version 18.0 software (IBM Inc., Chicago, IL, USA), with a P-value of less than 0.05 indicating statistical significance.

### Validation With an Independent Colorectal Cancer Dataset

In order to validate the results obtained for the TCGA dataset, we downloaded an independent dataset of CRC from the Gene Expression Omnibus (GEO) database, and subsequently compared the mean methylation of *NAT1* and that of each CpG site in different tissues, with a P-value of less than 0.05 indicating statistical significance.

## Results

### *N*-Acetyltransferase 1 mRNA Expression and Methylation in Cancers

**Figure 1:**
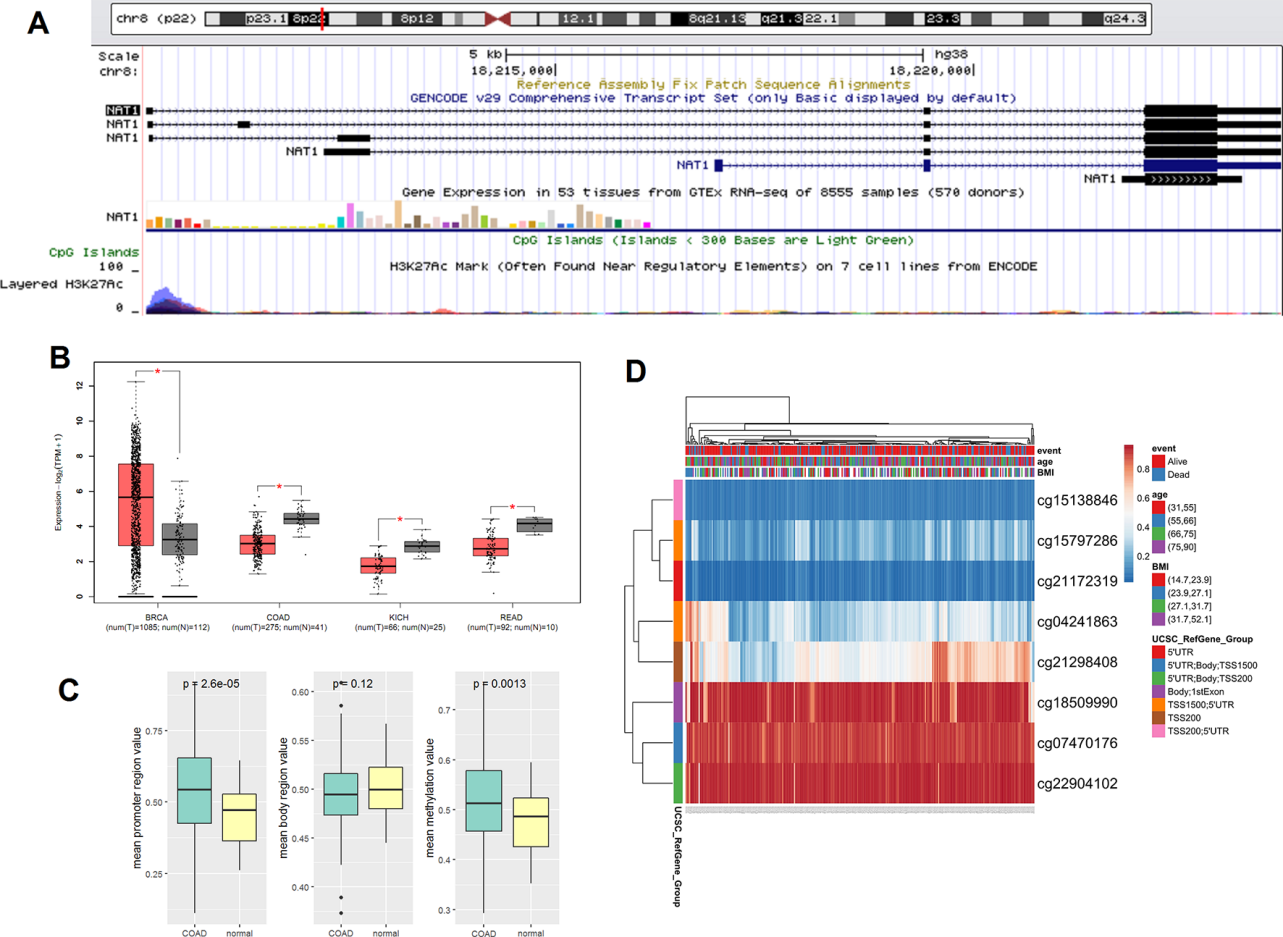
**(A)** Genomic position and functional annotation of *N*-acetyltransferase 1 (*NAT1*) gene from the University of California Santa Cruz genome browser; **(B)** Comparison of *NAT1* mRNA in cancers and corresponding normal tissues; **(C)** The mean methylation of *NAT1* in promoter region of COAD and normal tissues; **(D)** Heatmap of methylation CpG sites of *NAT1* in COAD. *p < 0.05.

The sequences of the *NAT1* gene CpG sites are presented in **Figure 1A**. As shown in **Figure 1B**, analysis of data from the TCGA database indicated that the expression of *NAT1* mRNA was markedly decreased in COAD, kidney chromophobe, and rectum adenocarcinoma tissues compared with the corresponding normal tissues, but increased in those of breast cancer (P < 0.05). The mean methylation of *NAT1* and promoter region CpG sites was markedly elevated in COAD tissues compared with that in normal colon tissues, although no significant differences were detected for the gene coding sequence (**Figure 1C**). The heatmap graph shown in **Figure 1D** shows the methylation of the eight CpG sites in COAD, which was visualized using the Clustvis web tool (Metsalu and Vilo, 2015). **Table 1** lists the names and locations of the eight CpG sites in the promoter region of *NAT1*.

**Table 1:** CpG sites of *N*-acetyltransferase 1 (*NAT1*) in the promoter region.

No.	Name	Location of promoter region
1	cg04241863	TSS1500; 5’UTR
2	cg07470176	5’UTR; Body; TSS1500
3	cg15138846	TSS200; 5’UTR
4	cg15797286	TSS1500; 5’UTR
5	cg18509990	Body; 1stExon
6	cg21172319	5’UTR
7	cg21298408	TSS200
8	cg22904102	5’UTR; Body; TSS200

### Correlation Analysis of *N*-Acetyltransferase 1 mRNA Expression and Methylation

**Figure 2:**
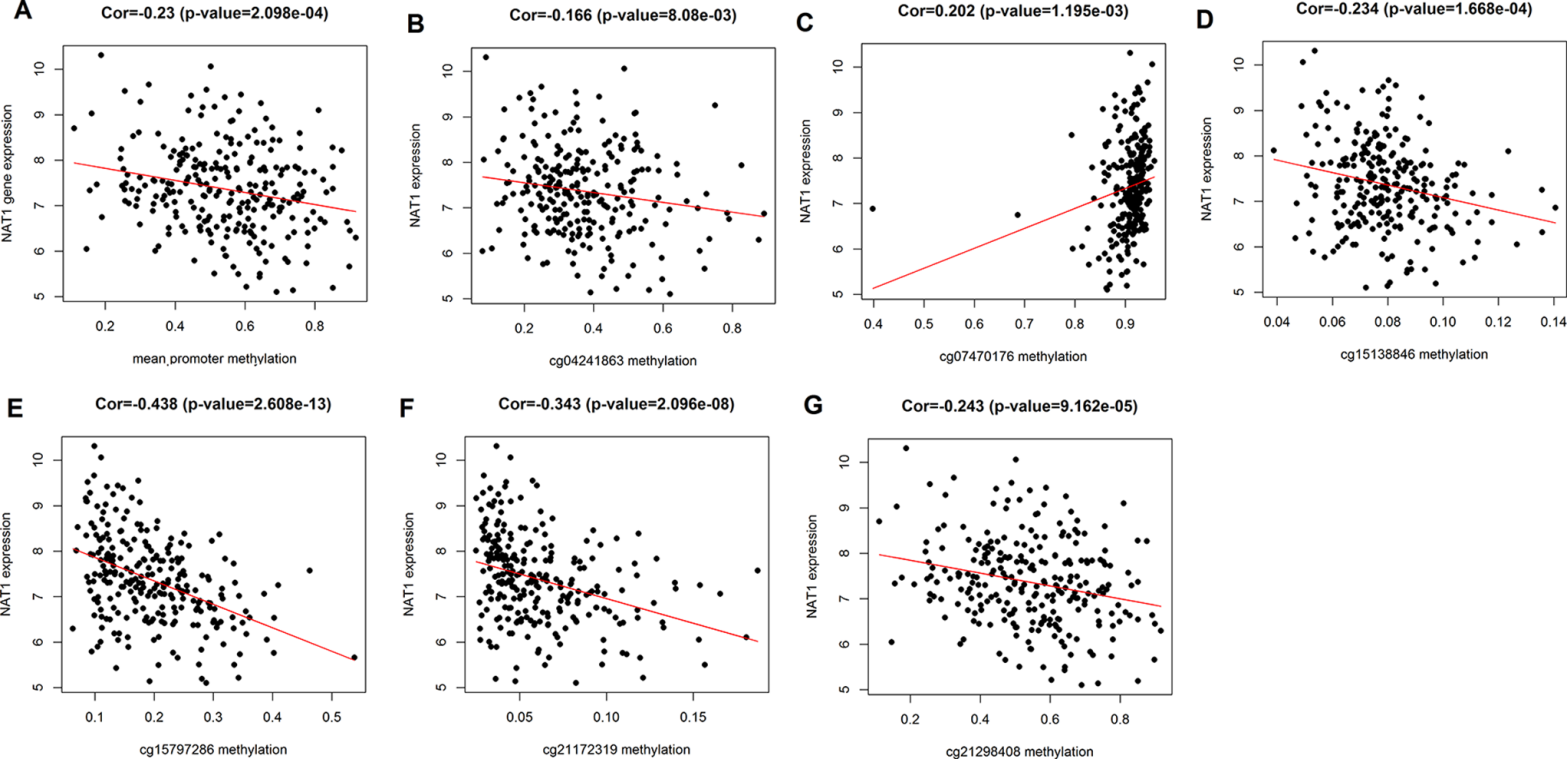
**(A)** Correlation analysis of *N*-acetyltransferase 1 (*NAT1*) mRNA with the mean methylation of promoter region; **(B**–**G)** Correlation analysis of *NAT1* mRNA with cg04241863, cg07470176, cg15138846, cg15797286, cg21172319, cg21298408.

We initially analyzed the correlation between *NAT1* mRNA expression and mean methylation of the promoter region, and accordingly found that *NAT1* mRNA expression was significantly negatively correlated with promoter methylation (P < 0.001) (**Figure 2A**), thereby indicating that methylation of the *NAT1* promoter region has a significant influence on the expression of *NAT1* mRNA. We subsequently conducted correlation analyses for associations between *NAT1* mRNA expression and methylation of each of the eight CpG sites, which revealed that five CpG sites (cg04241863, cg15138846, cg15797286, cg21172319, and cg21298408) were negatively correlated with the expression of *NAT1* mRNA in COAD (P < 0.05), whereas cg07470176 was positively correlated with *NAT1* mRNA expression (P < 0.05), and the remaining two CpG sites showed no significant correlation (P > 0.05) (**Figures 2B–G**).

### Analysis of Colon Adenocarcinoma Patient Survival Related to *N*-Acetyltransferase 1 CpG Site Methylation and Gene Expression

**Figure 3:**
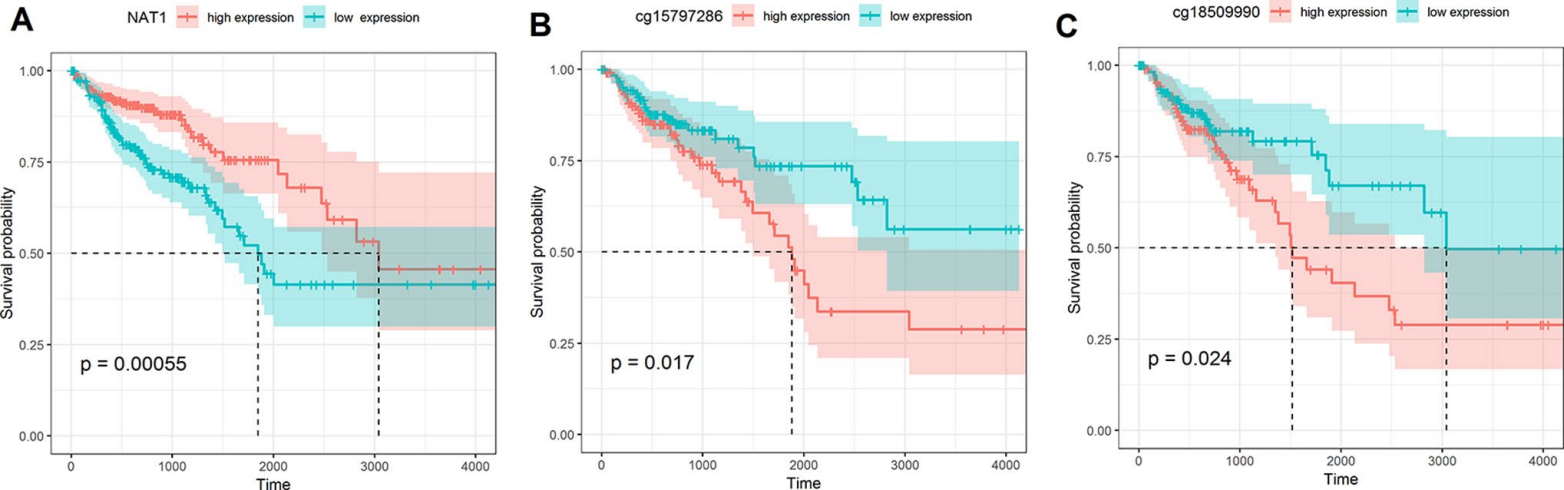
**(A)** Kaplan–Meier curve of *N*-acetyltransferase 1 (*NAT1*) mRNA expression in COAD; **(B)** Kaplan–Meier curve of cg15797286 in COAD; **(C)** Kaplan–Meier curve of cg18509990 in COAD.

Using the median value as a cut-off, we found that compared with a high expression of *NAT1* mRNA, a low expression was associated with a shorter OS in COAD patients. Subsequently, we examined the association between the methylation of individual *NAT1* promoter CpG sites and OS, and accordingly found that high methylation of two CpG sites in particular (cg15797286 and cg18509990) was associated with a shorter OS compared with those characterized by low methylation (**Figures 3B, C**). Consistently, Cox regression analysis revealed that methylation at these two CpG sites independently predicted the prognosis of patients with COAD (P < 0.05) (**Table 2**).

**Table 2:** *N*-acetyltransferase 1 (*NAT1*) CpG sites methylation associated with overall survival in colon adenocarcinoma (COAD).

CpG sites	HR	95%CI	P-value
cg04241863	1.06	0.66–1.71	0.790
cg07470176	0.82	0.51–1.34	0.444
cg15138846	0.85	0.53–1.38	0.525
cg15797286	1.72	1.05–2.80	0.028
cg18509990	1.78	1.09–2.91	0.021
cg21172319	1.49	0.93–2.40	0.095
cg21298408	1.49	0.92–2.40	0.100
cg22904102	0.78	0.46–1.33	0.376

### Analysis of Colon Adenocarcinoma Patient Survival Based on a Combination of *N*-Acetyltransferase 1 mRNA Expression and CpG Site Methylation

**Figure 4:**
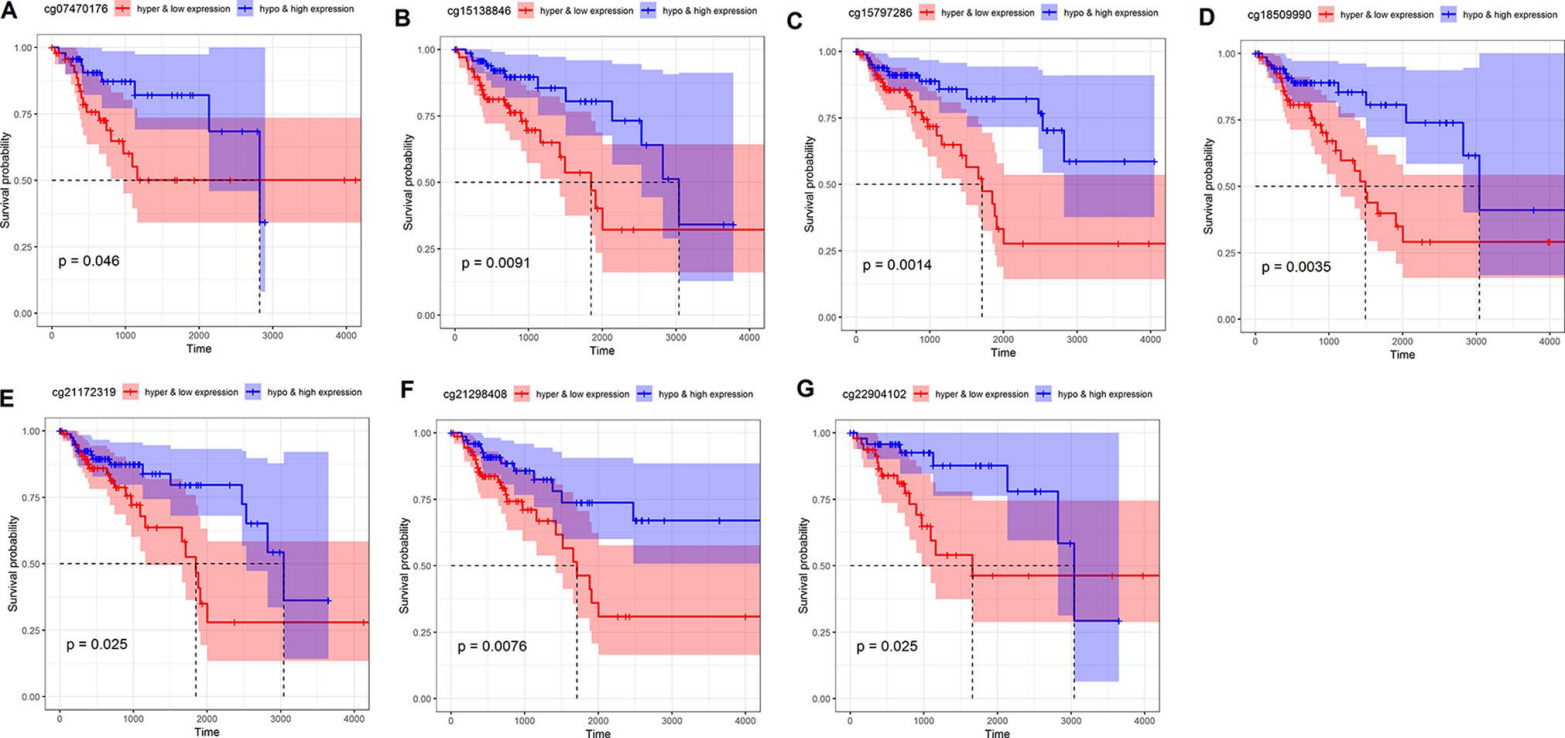
Kaplan–Meier curve of combination of *N*-acetyltransferase 1 (*NAT1*) mRNA with CpG sites in COAD. (**A**) *NAT1* mRNA with cg07470176; **(B)** *NAT1* mRNA with cg15138846; **(C)** *NAT1* mRNA with cg15797286; **(D)** *NAT1* mRNA with cg18509990; **(E)** *NAT1* mRNA with cg21172319; **(F)** *NAT1* mRNA with cg21298408; **(G)** *NAT1* mRNA with cg22904102.

In order to examine further indicators that could be used to predict the prognosis of COAD patients, we performed survival analysis based on combinations of *NAT1* mRNA expression and methylation of each CpG site, and accordingly found that seven such combinations were significantly associated with OS in COAD patients (P < 0.05) (**Figures 4A–G**). Cox regression analysis revealed that six of the seven combinations were independent predictive indicators in patients with COAD (P < 0.05) **Table 3**.

**Table 3:** Survival analysis of *N*-acetyltransferase 1 (*NAT1*) CpG sites combine with mRNA expression in colon adenocarcinoma (COAD).

Gene	CpG sites	HR	95%CI	P-value
*NAT1*	cg04241863	1.09	0.12–1.99	0.096
*NAT1*	cg07470176	1.51	1.13–2.03	0.006
*NAT1*	cg15138846	0.63	0.44–0.90	0.012
*NAT1*	cg15797286	0.59	0.42–0.83	0.002
*NAT1*	cg18509990	0.61	0.43–0.86	0.005
*NAT1*	cg21172319	0.69	0.50–0.96	0.028
*NAT1*	cg21298408	0.63	0.45–0.90	0.010
*NAT1*	cg22904102	1.04	0.18–2.29	0.103

### Association Between *N*-Acetyltransferase 1 mRNA Expression and CpG Site Methylation and the Clinicopathological Features of Colon Adenocarcinoma

In order to examine the association between *NAT1* mRNA expression and CpG site methylation and the clinicopathological features of COAD, we selected *NAT1* mRNA and the two CpG sites (cg15797286 and cg18509990) showing significant associations with the OS of COAD patients. We observed that *NAT1* mRNA expression was associated with the T stage, N stage, and clinical stage of COAD, and that similar results were obtained for cg15797286 (P < 0.05). In contrast, we detected no associations between cg18509990 and the clinicopathological features of COAD (P > 0.05) (**Table 4**).

**Table 4:** Association of *N*-acetyltransferase 1 (*NAT1*) mRNA and two CpG sites with clinicopathological features of colon adenocarcinoma (COAD).

	mRNA	P-value	cg15797286	p-value	cg18509990	P-value
Age		0.072		0.006		0.785
>60	7.58 ± 1.04		0.19 ± 0.08		0.91 ± 0.08	
≤60	7.34 ± 1.01		0.22 ± 0.09		0.92 ± 0.06	
Gender		0.111		0.909		0.406
Male	7.42 ± 1.04		0.20 ± 0.09		0.92 ± 0.07	
Female	7.62 ± 1.03		0.20 ± 0.08		0.91 ± 0.08	
T stage		0.022		0.024		0.877
T1 + T2	7.80 ± 0.95		0.17 ± 0.08		0.91 ± 0.06	
T3 + T4	7.44 ± 1.05		0.20 ± 0.08		0.91 ± 0.08	
N stage		<0.001		0.001		0.106
N0	7.76 ± 1.013		0.18 ± 0.08		0.91 ± 0.08	
N1 + N2	7.14 ± 0.97		0.22 ± 0.08		0.92 ± 0.06	
M stage		0.761		0.625		0.742
M0	7.52 ± 1.02		0.19 ± 0.08		0.91 ± 0.07	
M1	7.48 ± 1.08		0.20 ± 0.08		0.92 ± 0.08	
Clinical stage		<0.001		0.001		0.116
I + II	7.77 ± 0.99		0.18 ± 0.08		0.91 ± 0.08	
III + IV	7.18 ± 1.00		0.21 ± 0.08		0.92 ± 0.07	

### Validation of *N*-Acetyltransferase 1 CpG Site Methylation in Colon Cancer Using a Gene Expression Omnibus Dataset

**Figure 5:**
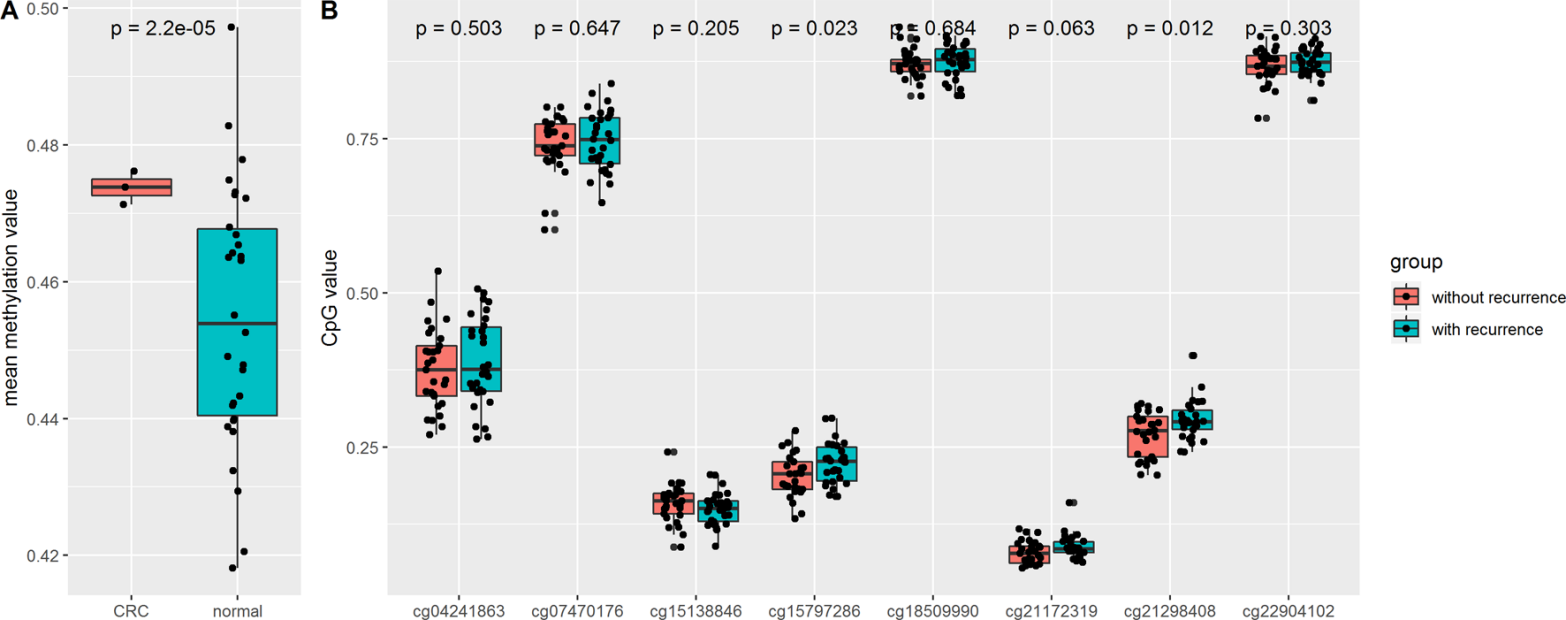
**(A)** Comparison of mean CpG value of *N*-acetyltransferase 1 (*NAT1*) in colorectal adenomas and normal tissues; **(B)** Comparison of methylation of CpG sites in colorectal adenomas and normal tissues.

In order to validate the aforementioned results based on analysis of a TCGA dataset, we downloaded an independent CRC dataset from the GEO database (GSE128067), which included non-recurrent colorectal adenoma (n = 30), recurrent colorectal adenoma (n = 29), and matched pair (n = 10) samples. The results showed that the mean methylation of the eight CpG sites was significantly increased in colorectal adenomas compared with normal tissues. We also found that compared with non-recurrent colorectal adenomas, the cg15797286 and cg21298408 sites in recurrent colorectal adenomas were characterized by higher levels of methylation (**Figure 5**).

## Discussion

DNA methylation is a vital type of epigenetic modification that is related to the regulation of gene expression and alternative gene splicing (Jones and Baylin, 2007), and is also implicated in the pathogenesis of numerous diseases, including various cancers (Michalak et al., 2019; Mahmood and Rabbani, 2019). CpG sites are located within or near the promoter regions of genes, and the aberrant methylation of these sites often confers a hypermethylation status and causes gene silencing (Wang et al., 2018). Importantly, DNA methylation is a chemically stable modification and can be detected at relatively low cost, and is therefore regarded as a promising type of non-invasive biomarker that can be used in the diagnosis and prognosis of cancers (Gloss and Samimi, 2014; Chen et al., 2014). To date, studies have identified the methylation of several genes, including *Claudin11* (Lee et al., 2016), *APC2* (Butcher and Minchin, 2012), and *ZNF331* (Liu et al., 2015), that are considered to be potential epigenetic biomarkers for the diagnosis and prognosis of CRC. In addition, a recent study showed that EREG/AREG methylation is associated with the efficacy of anti-EGFR therapy for CRC (Lee et al., 2016).

The *NAT1* gene, located on chromosome 8p22, encodes one of two human enzymes that are known to metabolize arylamine- and hydrazine-type drugs (Butcher and Minchin, 2012), and in this regard, a previous study has reported that low *NAT1* expression resulted in a distinctly poor response to chemotherapy in breast cancer patients (Minchin and Butcher, 2018). In COAD, *NAT1* has been found to be enriched in the caffeine metabolism pathway (Liu et al., 2015), and inhibition of its expression by curcumin has been demonstrated in COAD cells (Chen et al., 2003). In the present study, we found that the expression of *NAT1* mRNA was significantly reduced in COAD tissues compared with the corresponding normal tissues, which contrast with observations for the methylation of *NAT1*. Correlation analysis showed that mean methylation in the promoter region of *NAT1* was negatively correlated with *NAT1* mRNA expression, suggesting that *NAT1* may function as a tumor suppressor gene, and that a high level of methylation could reduce the mRNA expression of this gene in COAD tissues. These findings thus reveal that methylation of *NAT1* is associated with the development of COAD, and could thus serve as a potential biomarker for COAD development.

To further evaluate the effect of *NAT1* methylation on COAD, we analyzed the correlation between *NAT1* mRNA expression and the methylation of promoter CpG sites, and found that methylation of five of the eight CpG sites in the *NAT1* promoter was negatively correlated with *NAT1* mRNA expression, indicating that these five CpG sites may play regulatory roles in the expression of *NAT1* mRNA. We subsequently assessed the prognostic value of all eight CpG sites, and found that only two of the sites were associated with the OS of COAD patients. Given that previous studies have indicated that joint survival analysis based on a combination of data relating to the methylation and mRNA expression of genes can be used to identify candidate prognostic biomarkers (Gao et al., 2018; Lu et al., 2019), we also combined these data in the present study to determine the influence on patient survival. We accordingly found that combinations of *NAT1* mRNA expression with six of the eight CpG sites were significantly correlated with the prognosis of COAD patients. Thus, our results indicate that *NAT1* mRNA expression, *NAT1* methylation, and their combination have potential utility in predicting COAD patient survival.

A number of previous studies have reported the clinical significance of gene methylation in relation to COAD. For example, Ogino et al. (Ogino et al., 2009) investigated the methylation status of 649 COAD patients in two independent cohort studies, and quantified DNA methylation in the CpG island methylator phenotype (CIMP)-specific promoters of eight genes (*CACNA1G*, *CDKN2A*, *CRABP1*, *IGF2*, *MLH1*, *NEUROG1*, *RUNX3*, and *SOCS1*). They found that patients with CIMP-high cancers experienced a significantly low COAD-specific mortality, and that CIMP-high tumors were associated with a significant reduction in COAD-specific mortality, regardless of both MSI and BRAF status. Yagi et al. (Yagi et al., 2010) identified the occurrence of three methylation epigenotypes (high-, intermediate-, and low-methylation) in CRC, and found that the high-methylation epigenotype was correlated with MSI-high and BRAF-mutation(+), whereas KRAS-mutation(+) CRC associated with an intermediate-methylation epigenotype showed worse prognosis. These findings thus provided evidence that gene methylation is associated with the clinical significance and prognosis of COAD.

Nevertheless, although previous studies have demonstrated that the methylation status of several genes is associated with the development of COAD, there is currently limited data available regarding the clinical significance of *NAT1* expression and methylation in COAD. In this study, we found that *NAT1* mRNA expression was associated with tumor T stage, N stage, and clinical stage, but not M stage. We also evaluated the clinical significance of two CpG sites (cg15797286 and cg18509990), and detected similar associations with respect to cg15797286, although not cg18509990. Interestingly, we observed the trend that the association of *NAT1* mRNA expression with each of the evaluated clinicopathological feature was opposite to that of the association of these features with cg15797286 methylation, which further confirmed the correlation between *NAT1* mRNA and cg15797286. These results also indicated that *NAT1* mRNA expression and cg15797286 methylation are associated with the development of COAD. Moreover, in our analysis of a validated dataset, we found that the methylation of cg15797286 was increased in recurrent CRC, which further verified the association between cg15797286 and the development of COAD. Compared with previous studies that have examined the association of gene methylation with COAD (Ogino et al., 2009; Yagi et al., 2010), in the present study, we focused on a single variant of *NAT1*, and found that cg15797286 was associated with the clinical stage and survival of COAD patients, which have been reported previously.

To the best of our knowledge, this is the first study that has investigated the prognostic value and clinical significance of *NAT1* methylation in COAD. Moreover, we provide the first evidence that the cg15797286 site in the *NAT1* promoter region is closely related to the mRNA expression of this gene, as well as to COAD stage and patient survival. Despite these important findings, however, the study does have certain limitations that should be noted. First, the sample size of the validated data obtained from the GEO database was small, and we were unable to distinguish between COAD and rectum adenocarcinoma in the sample data. Thus, we could not verify our results with regard to COAD. Second, due to a lack of survival data for COAD, we were unable to verify the prognostic value of *NAT1* methylation in the validated dataset. Third, the biological function of the *NAT1* gene and CpG sites and their relationships with COAD remain largely unknown. Consequently, further study of a large COAD cohort is warranted to verify the prognostic value of *NAT1* methylation, and *in vivo* and *in vitro* experiments are needed in order to elucidate the biological function of *NAT1* and its CpG sites with respect to COAD.

## Conclusion

In this study, we demonstrate that the *NAT1* gene is characterized by a hypermethylated status in COAD, and that the cg15797286 site in the *NAT1* promoter is closely related to the development and prognosis of COAD. These findings indicate that the DNA methylation of *NAT1* could serve as a prognostic marker and potential therapeutic target for COAD. However, due to the aforementioned limitations, further studies are warranted to validate our results.

## Data Availability Statement

Publicly available datasets were used in this study. This data can be found here: http://gdac.broadinstitute.org/. 

## Author Contributions

Study concept and design: CS, B-LH; Collection and assembly of data: CS, K-ZL, LL and J-LL; Data analysis and interpretation: K-ZL, Y-PT; Manuscript writing and review: All authors.

## Funding

This study was partially supported by research funding from the National Natural Science Foundation (No. 81260083), Natural Science Foundation of Guangxi (No. 2018JJA140136), and College Students Innovative Entrepreneurship Project of Guangxi (No. 201910598012; WLXSZX19039).

## Conflict of Interest

The authors declare that the research was conducted in the absence of any commercial or financial relationships that could be construed as a potential conflict of interest.

## Abbreviation

CRC, colorectal cancer; COAD, colon adenocarcinoma; *NAT1*, *N*-acetyltransferase 1; OS, overall survival; *APC2*, APC regulator of wnt signaling pathway 2; *ZNF331*, zinc finger protein 331; *HOXA2*, homeobox protein hox-A2; *PBX3*, PBX Homeobox 3.
