# Supplementary material for: Hypermethylation of N-Acetyltransferase 1 Is a Prognostic Biomarker in Colon Adenocarcinoma
Source: Front Genet. 2019 Nov 6;10:1097. doi: 10.3389/fgene.2019.01097 (PMC6851262; doi:10.3389/fgene.2019.01097)
Supplement: Supplementary file 1 [file DataSheet_1.pdf]

Supplemental Materials

Table S1 mRNA variants of *NAT1* and the CpG sites of *NAT1* methylation

mRNA variants	CpG sites
NM_000662	cg04149472; cg21363706; cg04241863; cg15797286; cg15138846; cg21172319
NM_001160170	cg04149472; cg21363706; cg04241863; cg15797286; cg15138846; cg21172319
NM_001160171	cg04149472; cg21363706; cg04241863; cg15797286; cg15138846; cg21172319
NM_001160172	cg04149472; cg21363706; cg04241863; cg15797286; cg15138846; cg21172319
NM_001160173	cg04149472; cg21363706; cg04241863; cg15797286; cg15138846; cg21172319
NM_001160174	cg07470176; cg09102997; cg22904102
NM_001160175	cg04149472; cg21363706; cg04241863; cg15797286; cg15138846; cg21172319
NM_001160176	cg04149472; cg21363706; cg04241863; cg15797286; cg15138846; cg21172319
NM_001160179	cg19721541; cg06450790; cg21298408; cg02041869

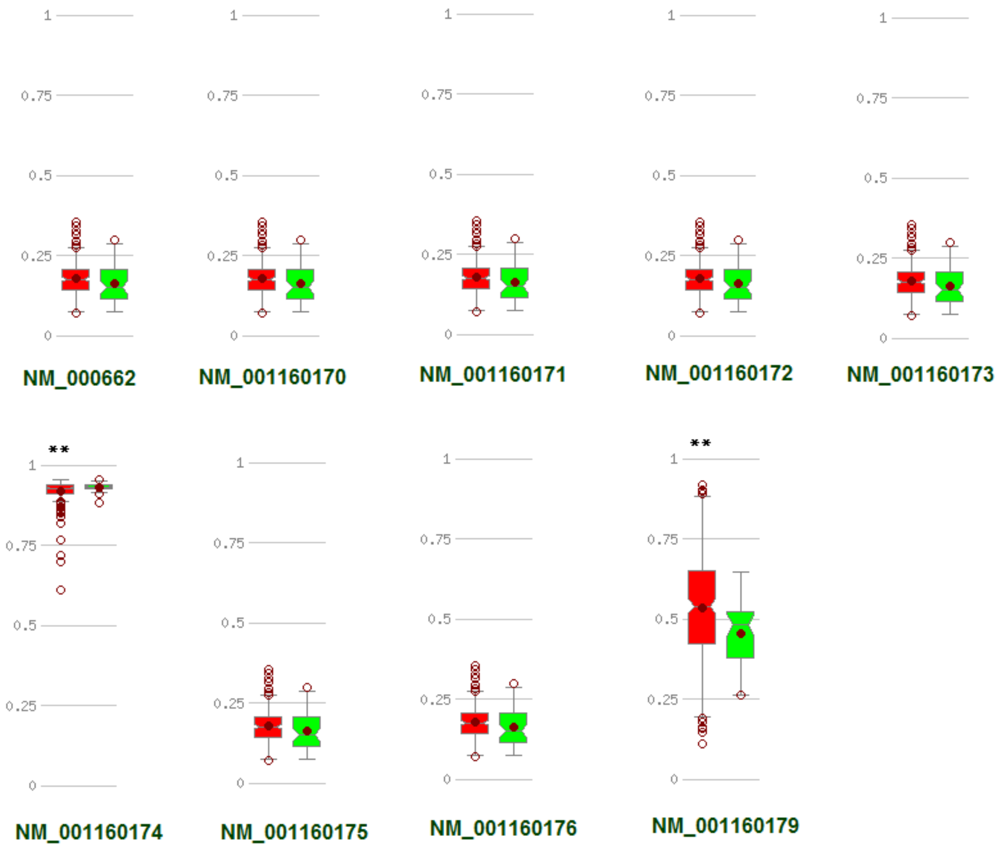

Figure S1 Comparison of methylation values of nine *NAT1* gene variants between tumor and normal tissue of COAD. Results were from MethHC website (http://methhc.mbc.nctu.edu.tw). Methylation of

NM_001160179 and NM_001160179 were significantly increased in COAD tissues compared with normal tissues. **P<0.001.
